# Strumal Carcinoid Presenting as Large Pelvic Mass: A Rare Case and Review of Literature

**DOI:** 10.7759/cureus.20494

**Published:** 2021-12-17

**Authors:** Sanobar Yasmeen Mohammed, Obainuju Mercy Anelo, Farhan Khan

**Affiliations:** 1 Department of Pathology and Laboratory Medicine, Orlando Regional Medical Center, Orlando, USA; 2 Department of Pathology and Laboratory Medicine, University of Tennessee Health Science Center, Memphis, USA

**Keywords:** insular pattern, monodermal teratoma, synaptophysin, ttf-1, postmenopausal, neuroendocrine, thyroid tissue, ovarian cyst, pelvic mass, strumal carcinoid

## Abstract

Strumal carcinoid is an unusual rare ovarian teratoma characterized by the presence of thyroid tissue with a carcinoid tumor. We report a case of a 60-year-old nulliparous woman, who presented with complaints of a decrease in appetite, urinary frequency, and left lower extremity edema. By ultrasound of the abdomen, a large multiloculated cystic lesion occupying almost the entire pelvis and measuring 24 x 14 x 20 cm with internal debris concerning either uterine or ovarian cystic carcinoma was seen. By MRI, it was confirmed to be an ovarian lesion. Labs revealed elevated cancer antigen 125 (CA125) of 105 U/ml and carcinoembryonic antigen (CEA) of 6.4 ng/ml. The patient underwent surgery and the intraoperative consultation confirmed teratoma with a neuroendocrine component. Grossly, it was a multicystic ovarian mass and on sectioning, it had partial solid and cystic areas with clear to mucoid fluid. Histopathology showed foci of ectopic thyroid tissue admixed with foci of well-differentiated neuroendocrine tumor, grade 1 (carcinoid) displaying insular and trabecular patterns consistent with the diagnosis of strumal carcinoid (monodermal teratoma). Thyroid transcription factor-1 (TTF-1) and thyroglobulin immunostains highlighted ectopic thyroid tissue and synaptophysin highlighted neuroendocrine component. Strumal carcinoids are almost invariably benign and pathologic staging is not warranted. Treatment of strumal carcinoid is salpingo-oophorectomy.

## Introduction

Strumal carcinoid is an ovarian teratoma accounting for 3% of all ovarian teratomas and is characterized by the presence of thyroid tissue with a carcinoid tumor. The carcinoid component is a well-differentiated neuroendocrine tumor with an excellent prognosis. Pre- and post-menopausal women have shown increased incidence. Symptoms range from a benign pelvic mass with mass-compression effects to carcinoid syndrome. The various histopathological patterns in the presentation have been a point of debate for the diagnosis. The occurrence of papillary thyroid carcinoma in the strumal carcinoid is a growing concern, hence patients should be diligently managed [1,2].

The abstract was previously presented as a meeting poster at the 2021 College of American Pathologists (CAP) Annual Scientific Meeting CAP21 held in Chicago on September 25, 2021, and also published in the Archives of Pathology & Laboratory Medicine, vol. 145, issue 9, September 2021. (https://meridian.allenpress.com/aplm/article/145/9/e2/469635/Abstracts-and-Case-Studies-for-the-College-of)

## Case presentation

**Figure 1:**
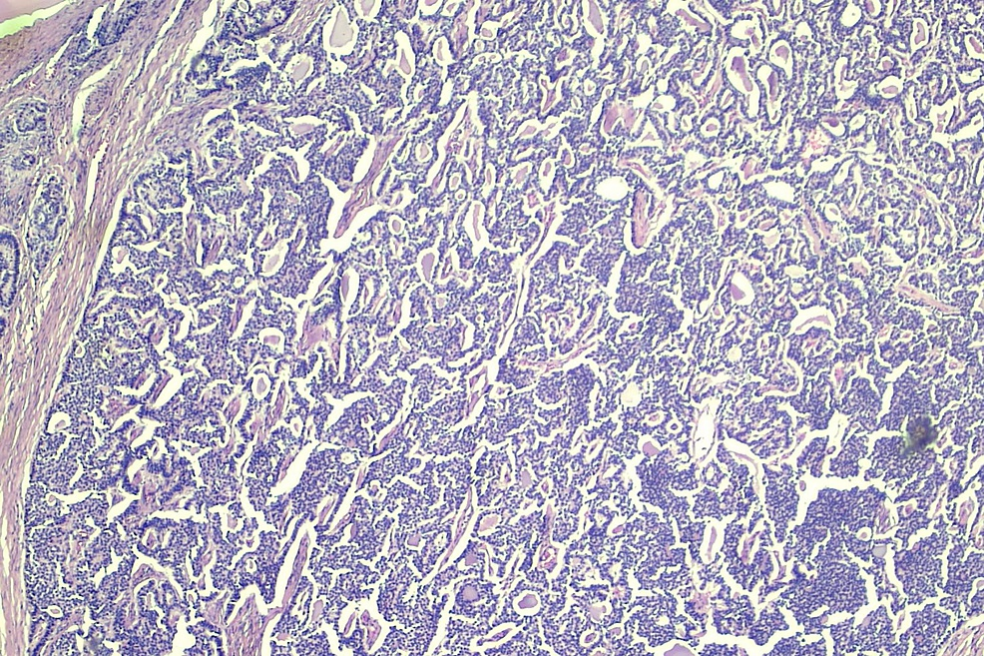
Hematoxylin and eosin illustrates both the carcinoid and thyroid component of strumal carcinoid (4x)

A 60-year-old, nulliparous per clinical records, post-menopausal Caucasian woman (the patient attained menopause at 51 years) presented with abdominal pain, loss of appetite, urinary frequency, and left lower extremity edema. Physical examination was pertinent for epigastric tenderness, palpable mass in the umbilical area, and the presence of an umbilical hernia. Abdominal ultrasound done at an outside facility revealed a large multiloculated cystic lesion occupying almost the entire pelvis measuring 24 x 14 x 20 cm containing internal debris concerning either uterine or ovarian cystic carcinoma. Magnetic resonance imaging (MRI) demonstrated a large complex cystic mass measuring approximately 29 x 14 x 21 cm with a solid enhancing internal component consistent with ovarian tumor (the imaging was done at an outside facility and only notes were available for the case report). Laboratory data were pertinent for cancer antigen 125 (CA125) of 105 U/ml (reference range <46 U/ml) and carcinoembryonic antigen (CEA) of 6.4 ng/ml (reference range < 2.5 ng/ml). The patient underwent a total abdominal hysterectomy with left salpingo-oophorectomy, omentectomy, pelvic aortic lymph node biopsy, umbilical hernia repair, and optimal peritoneal tumor debulking was performed. Intraoperative consultation on frozen confirmed teratoma with the neuroendocrine component. The gross examination of the left ovary (17 x 13 x 4 cm) revealed a partially solid (40%) and partially cystic mass (60%) containing clear, bloody, and mucoid fluid in different cysts. Histopathological examination of the left ovary showed foci of ectopic thyroid tissue with colloid-filled follicles admixed with foci of well-differentiated neuroendocrine tumor, grade 1 (carcinoid) consistent with the diagnosis of strumal carcinoid (monodermal teratoma) (Figure 1).

**Figure 2:**
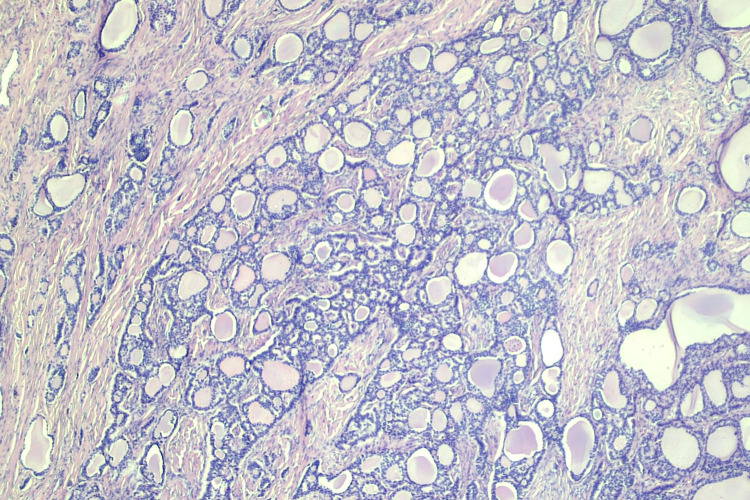
Hematoxylin and eosin section illustrates the thyroid component in strumal carcinoid (4x)

**Figure 3:**
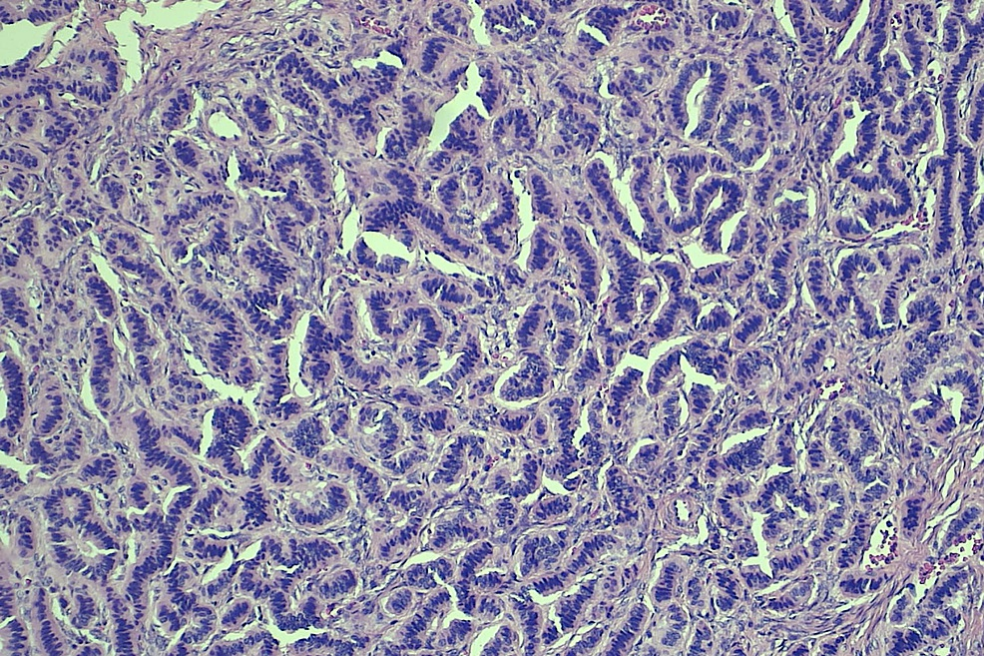
Hematoxylin and eosin section illustrates the neuroendocrine component in insular pattern (4x)

The micro and macro follicles of the thyroid tissue are lined by benign cuboidal to flat epithelium mimicking the normal thyroid parenchyma (Figure 2). The carcinoid component was arranged in insular and trabecular patterns with dense stromal hyalinization (Figure 3).

**Figure 4:**
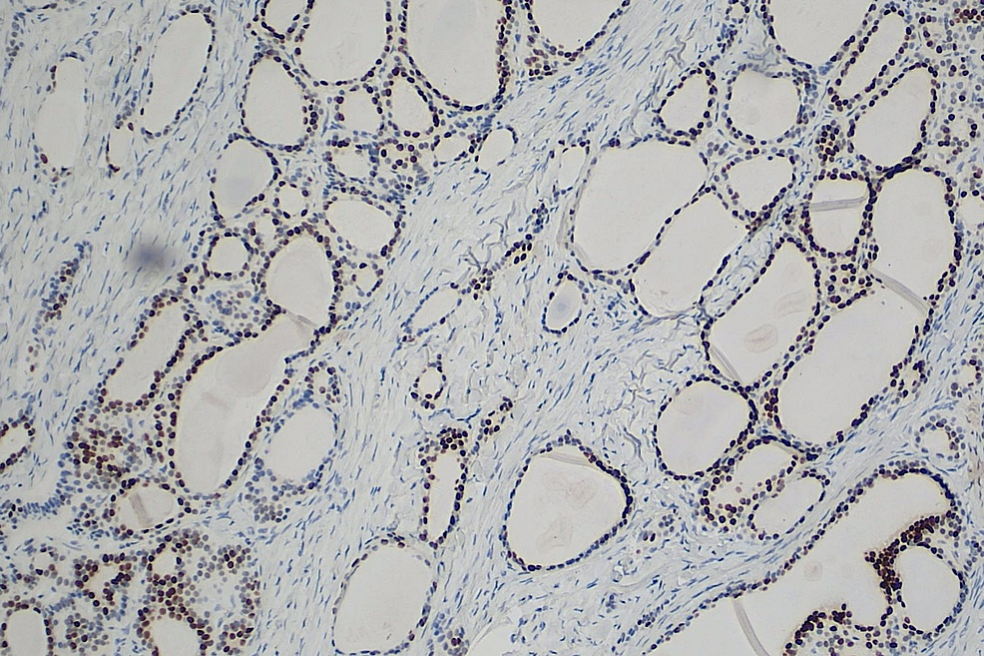
TTF-1 nuclear stain highlights the thyroid follicular cells (10x) TTF-1: thyroid transcription factor-1

**Figure 5:**
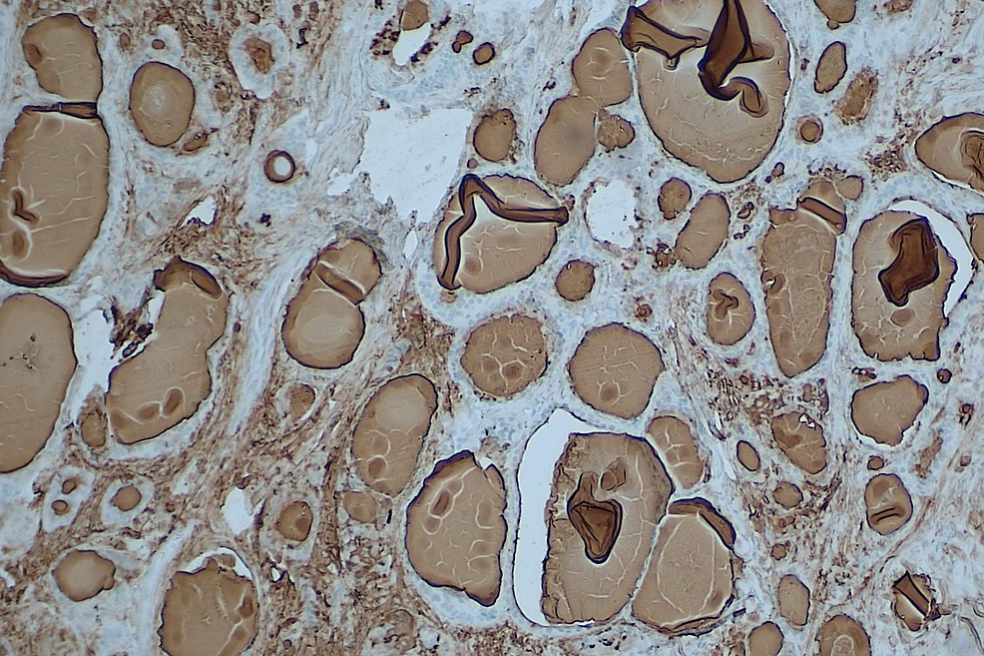
Thyroglobulin highlights the thyroid tissue (10x)

**Figure 6:**
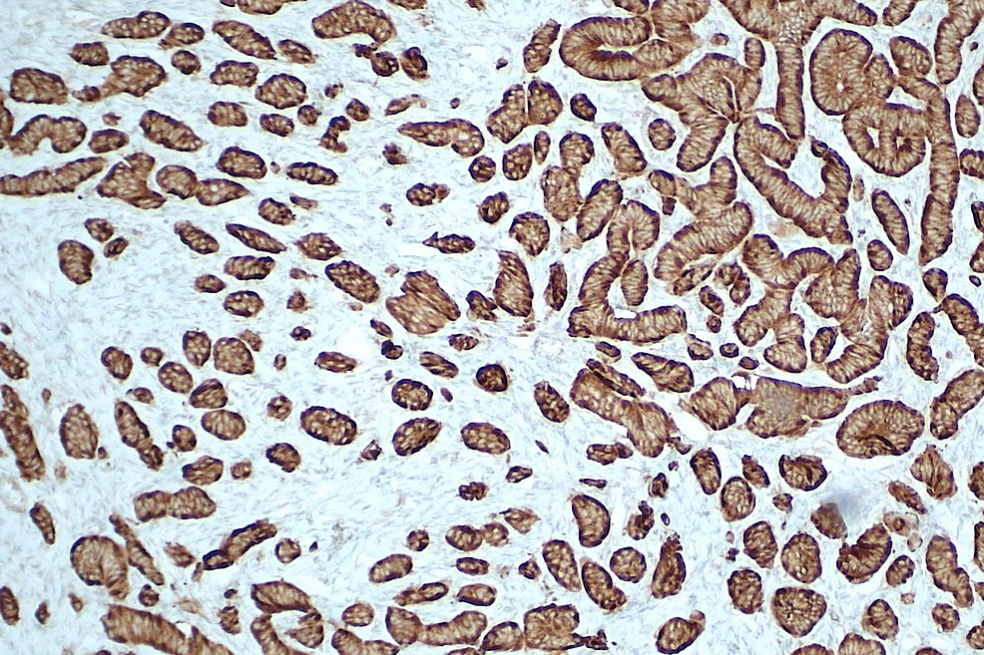
Synaptophysin highlights the carcinoid tissue (10x)

By immunohistochemistry (IHC) the thyroid parenchyma displayed nuclear positivity by expression of thyroid transcription factor-1 (TTF-1) (Figure 4). Cytoplasmic thyroglobulin also highlighted the thyroid tissue (Figure 5). The neuroendocrine component was positive for synaptophysin and chromogranin immunostain (Figure 6). Histopathological examination and confirmation of the diagnosis with the IHC guided the physicians in management. The patient recovered well after surgery and was discharged thereafter.

## Discussion

Strumal carcinoid is the most common form of monodermal teratomas arising in the ovary. Nearly 3% of teratomas account for strumal carcinoid type which is an admixture of benign thyroid tissue and carcinoid. Strumal carcinoids can be pure and sometimes in association with mature teratomas, granulosa tumors, and mucinous cystadenomas of the ovary [1,2]. These are reported mainly in pre- and post-menopausal women with a peak in the fifth decade of life [1,3-8].

The histogenesis of its origin is being debated as it is mostly considered as a component of the teratoma as a germ cell origin. The carcinoid component is native to the ovary and less likely to be a metastatic component from the gastrointestinal tract [4-8]. There is also a theory about hybrid cells differentiating into thyroid follicular cells and hindgut neuroendocrine cells [9,10], and the carcinoid arises from neuroendocrine cells of the genital tract [3]. Signs and symptoms can vary from mild complaints like constipation, pelvic mass to rare symptoms from a functional thyroid tissue, or a typical carcinoid. Very rare patients presented with pleural effusion along with pelvic mass-Meigs syndrome or symptoms of virilism [1,3,4,7,11,12]. Meigs syndrome is mostly associated with thecoma or a fibroma. Symptoms of virilism were the results of excess secretion of steroidogenic hormones in rare patients which eventually resolved with removal of the mass. Davis et al. have done a detailed review of literature on primary carcinoid of the ovary and reported only four cases of strumal carcinoid in his study presenting with a pelvic mass and did well with surgery only with no adverse outcomes [2].

The primary strumal carcinoid of the ovary is mostly unilateral [6,7]. Grossly it's a firm, solid tumor sometimes cystic and gray-white to yellow on cut section [1,3,7]. They can also appear as thickened lining of cyst wall and also as a component of other neoplasms [1,3-5]. According to WHO, carcinoids are of four histological types which include insular, trabecular, strumal, and mucinous [1,3,4,8,12]. Insular is the most common type followed by strumal [7,10]. A well-differentiated neuroendocrine tumor is seen. The insular pattern consists of solid nests separated by fibrous stroma, cells with a moderate amount of cytoplasm, and central nuclei. The trabecular form has long ribbons, cords, or thin trabeculae arrangement of cells in which cells have a moderate amount of cytoplasm and nuclei which are arranged parallel to each other and perpendicular to the basement membrane [4,10]. Strumal can either be insular or trabecular or both forms intermixed with thyroid tissue. Well-differentiated mucinous carcinoid is lined by intestinal epithelium and arranged in small glands floating in pools of mucin [1,3,5,8]. Thyroid tissue is represented in micro or macro follicles with benign cuboidal follicular cells and colloids. But sometimes colloid goiter toxic changes with hyperplastic follicular cells, thyroid adenoma, and rarely carcinoma can also be present [1,12]. The neuroendocrine tissue of strumal carcinoid is positive for chromogranin, synaptophysin, cluster of differentiation (CD)56, neuron-specific enolase (NSE), and serotonin [1,3,5]. The thyroidal tissue is positive for thyroglobulin, TTF-1 [1,3,5,8].

The differential diagnosis for strumal carcinoid can include granulosa cell tumor (microfollicular), benign Brenner tumor, metastatic carcinoid to the ovary, or metastatic thyroid carcinoma to the ovary [7,8,12], an admixture of thyroid and carcinoid components may help to differentiate from pure thyroid carcinoma metastasis [7]. Metastatic carcinoids are mostly bilateral compared to primary carcinoids of the ovary which are unilateral [7]. Carcinoids have also been found to be a component of sex cord-stromal tumors, Sertoli-Leydig cell tumors [1,3,4,8]. Call exner bodies of granulosa cell tumor can resemble carcinoid acinus [4].

Strumal carcinoids are mostly confined to the ovary, are indolent, and resolve by surgical treatment by salpingo-oophorectomy [6,8,11,12]. Rare cases were reported with widespread metastasis which needs to be treated with radical resection followed by chemotherapy [6]. Our patient survived surgery and is doing well. Carcinoid component in strumal carcinoid is considered to be native to the ovary, most probably arising from differentiation of pluripotent germ cells not a metastasis from the gastrointestinal tract (GI). Clinical and radiological correlation is required for the treatment modality. In most cases, metastasis from the GI tract is less likely. Cancers are rare in strumal carcinoids. Sometimes papillary carcinoma of the thyroid (PTC) can arise followed by follicular carcinoma and even rare insular carcinoma are reported [1,5,7,8,12]. A lot of literature has been written about differentiating papillary carcinoma arising in the thyroid component of struma ovarii and metastatic PTC from thyroid primary to the ovary [5]. In the absence of thyroid carcinoma, there is no clear advantage of radiotherapy.

## Conclusions

Strumal carcinoids are rare but the most common variant of monodermal teratoma which can arise in the ovary. Benign thyroid tissue and well-differentiated neuroendocrine tumor are admixed with various architectural patterns and stromal hyalinization. Strumal carcinoids are unilateral benign tumors and pathologic staging is not warranted. Surgery is the common mode of treatment for these patients. It is important to identify these tumors, especially during intraoperative frozen sections to avoid extensive surgery and staging.
